# Multiple independent transmission cycles of a tick-borne pathogen within a local host community

**DOI:** 10.1038/srep31273

**Published:** 2016-08-08

**Authors:** Maude Jacquot, David Abrial, Patrick Gasqui, Severine Bord, Maud Marsot, Sébastien Masseglia, Angélique Pion, Valérie Poux, Laurence Zilliox, Jean-Louis Chapuis, Gwenaël Vourc’h, Xavier Bailly

**Affiliations:** 1 INRA, UR346 Epidémiologie animale, Saint Genès Champanelle, France; 2 CNR des Borrelia, Hôpitaux Universitaires de Strasbourg, 6700 Strasbourg, France; 3 MNHN, Centre d’Ecologie et des Sciences de la Conservation, UMR 7204, Sorbonne Universités, MNHN, CNRS, UPMC, CP53, 61 rue Buffon, 75005 Paris, France

## Abstract

Many pathogens are maintained by multiple host species and involve multiple strains with potentially different phenotypic characteristics. Disentangling transmission patterns in such systems is often challenging, yet investigating how different host species contribute to transmission is crucial to properly assess and manage disease risk. We aim to reveal transmission cycles of bacteria within the *Borrelia burgdorferi* species complex, which include Lyme disease agents. We characterized *Borrelia* genotypes found in 488 infected *Ixodes ricinus* nymphs collected in the S**é**nart Forest located near Paris (France). These genotypes were compared to those observed in three sympatric species of small mammals and network analyses reveal four independent transmission cycles. Statistical modelling shows that two cycles involving chipmunks, an introduced species, and non-sampled host species such as birds, are responsible for the majority of tick infections. In contrast, the cycle involving native bank voles only accounts for a small proportion of infected ticks. Genotypes associated with the two primary transmission cycles were isolated from Lyme disease patients, confirming the epidemiological threat posed by these strains. Our work demonstrates that combining high-throughput sequence typing with networks tools and statistical modeling is a promising approach for characterizing transmission cycles of multi-host pathogens in complex ecological settings.

Many pathogens that infect humans are zoonotic—they are maintained in animal hosts^1^ —and a significant proportion of them is transmitted by vectors^2^. More than 70% of zoonotic infectious diseases have originated in wild species, and most are caused by pathogens that have multiple hosts^3^,^4^. These host species serve as reservoirs, maintaining and transmitting pathogens^5^,^6^. However, different pathogen genotypes can be transmitted at different rates to host and vector species and/or populations, which may result in the emergence of distinct transmission cycles and lead to disparities in the contributions of different host populations to disease risk.

As a consequence, understanding how the genetic diversity of zoonotic pathogens is partitioned among hosts and/or vectors is key to untangling pathogen circulation^7^. First, pathogen genotype frequencies within host and vector populations can vary according to the efficiency of pathogen transmission within and between populations^6^. Second, barriers to transmission as well as interactions among pathogen genotypes (*e.g.*, facilitation) can result in population-specific patterns of co-infection^8^. Third, spatial distributions of pathogen genotypes might vary as a result of the behavior of infected species^9^. Finally, by characterizing the genetics of the pathogens found in reservoir hosts, vectors, and humans, it is possible to study, and eventually mitigate, the human health risks posed by the different genotypes in circulation. The benefits are two-fold. Understanding pathogen diversity in vectors and humans may provide insight into the zoonotic potential of different pathogen genotypes. Identifying the reservoirs of zoonotic genotypes can help inform the development of preventive measures and thus reduce human health risks.

Over the last fifteen years, multilocus sequence typing (MLST) based on Sanger sequencing has been the “gold standard” when studying the genotypic diversity of bacterial pathogens^10^. However, if bacteria are not isolated beforehand, it is problematic to use this method to characterize the bacterial pathogens that form co-infections. Indeed, Sanger sequencing is not designed to generate accurate sequences when different genotypes are present and thus cannot provide a detailed description of the genetic diversity of pathogens participating in co-infections. Luckily, high-throughput sequencing approaches can now be used to circumvent these problems and more fully characterize pathogen genetic diversity in host and/or vector populations.

The *B. burgdorferi* species complex (*i.e.*, *B. burgdorferi* sensu lato [s.l.]) includes the infectious agent that causes Lyme disease, which is the most important vector-borne disease in the temperate zones of the Northern Hemisphere. This pathogen is an ideal study organism with which to explore the usefulness of high-throughput data in epidemiological settings. In Europe, the bacteria responsible for Lyme disease are transmitted by a generalist tick vector, *Ixodes ricinus*, to numerous vertebrate host species. As no vaccine is available for humans and late detection of the disease can entail prolonged treatment, disease control efforts focus on prevention. Even though human exposure driven by land use is important to accurately estimate human disease risk^11^, data obtained from questing ticks can provide a picture of how humans are exposed to the pathogens. Comparing the genotypes in circulation versus those associated with human cases of disease can reveal genotype-specific zoonotic potential, which is crucial since no methods for experimentally testing virulence in humans are available. Moreover, characterizing the contribution of each member of the transmission cycle to human Lyme disease risk could significantly advance the development of durable disease control strategies.

As culturing these bacteria is time consuming and not always successful, the epidemiological surveillance of Lyme disease agents has mainly involved the MLST typing of infected ticks, where bacteria are not isolated beforehand^12^. However, this method can be problematic when studying the genetic diversity of the *B. burgdorferi* species complex because co-infections within host species and ticks commonly occur^13^,^14^,^15^,^16^. A high-throughput sequencing protocol based on two loci has been developed for these bacteria^17^ but has never been used to characterize the genotypes circulating in ticks. The two loci are a chromosomal housekeeping gene used for classical multi-locus sequence typing and a plasmidic highly variable bacterial antigenic gene. These two loci, for which sequences from many strains are available, are suitable for phylogenetic analyses. Allele frequencies at the *ospC* gene, which encodes the outer surface protein C, differ among host species^18^; the same is true for the housekeeping gene, *rplB*^17^. Moreover, using hybridization methods, studies of co- infection patterns in tick species have found evidence that vertebrate host species structure genotype associations within ticks^19^. These observations raise questions on the actual contribution of each host species to the diversity carried by ticks and thus to human disease risk. In this context, combining sequencing data obtained from hosts and ticks would give a tremendous opportunity to provide further insight into the different reservoirs of *B. burgdorferi* s.l. genotypes.

An important part of establishing control measures is indeed estimating the contribution of each reservoir to the risk of humans being exposed to Lyme disease. Different models have been developed to characterize such contributions using genetic descriptions of circulating pathogens^20^,^21^. These models exploit differences in the frequency or transmission rates of different pathogen genotypes in different host species to estimate the proportion of infected vectors generated by each host species. To parameterize the most complex of these models, large amounts of data are required, such as the pathogen genotypes observed in questing ticks, the densities of the different host species, host-specific tick burdens, and the probability of a given genotype being transmitted from a specific host to a specific vector. Information on host contributions to *B. burgdorferi* s.l. genotype circulation is needed to understand human exposure dynamics, but in actuality only a few of the genotypes in circulation cause disease in humans^22^,^23^. Work comparing the genotypes found in both vectors and humans has indeed revealed that some genotypes show stronger zoonotic potential^24^. This information should be taken into account when optimizing preventive measures.

In this study, we used high-throughput sequencing to characterize the genetic diversity of *B. burgdorferi* s.l. bacteria found in 488 questing ticks sampled at a single study site. Using these data and previously gathered information about the genotypes circulating in different host species at this location, our aims were the following: i) to identify genotype transmission cycles by analyzing genotype occurrence patterns in different hosts and vectors; ii) to estimate the contributions of different host populations to the distribution and abundance of the different genotypes in ticks using a modeling approach; and iii) to determine whether any of the genotypes observed matched those associated with reported human cases. We discuss the relevance of our findings for the molecular epidemiology of the *B. burgdorferi* species complex and for other vector-borne diseases.

## Results

### Raw data

In total, we obtained 288,151 sequences that were assigned to individual ticks and deposited in the Sequence Read Archive (SRA) under the accession number SRP041191. Of those sequences, a total of 75,973 were assigned to the *rplB* gene and 144,604 were assigned to the *ospC* gene. For the 488 nymphs found to be infected, we obtained a mean of 155.7 *rplB* sequences and 296.3 *ospC* sequences per individual tick. Overall, we obtained sequences from 472 different nymphs; only a few individuals did not yield sequence data.

We also used 16,222 sequences obtained from small mammals that were deposited in the SRA under the accession number SRP032755. A mean of 33.2 *rplB* sequences and 37.9 *ospC* sequences were obtained from each individual host^17^.

### Genotype delineation

Using the nearest-neighbor classification algorithm, 39 and 82 genotypes were delineated from a total of 83,266 *rplB* sequences and 152,807 *ospC* sequences, respectively. We realized rarefaction analyses based on the resampling of raw sequences, for which sequencing efforts were comparable for ticks and hosts at the whole-dataset scale. In these analyses, we therefore considered a much less important sequencing effort for ticks than what we actually obtained. The results revealed certain patterns.

First, the relationship between the number of delineated genotypes and the number of sequences used quickly plateaued, which illustrated that we had captured most of the diversity of the species complex (Supplementary Fig. S3A). More importantly, when a high number of sequences were used, we did not observe a decrease in the number of delineated genotypes, which might have resulted if there had been clustering of divergent sequences due to an accumulation of sequencing errors.

Second, given that sequencing efforts were comparable, a larger number of genotypes were delineated in ticks than in hosts (Supplementary Fig. S3A). The mean number of genotypes per tick was slightly higher than that per host, but the variance in genotype number was similar (Supplementary Fig. S3B). This result means that the greater number of genotypes actually observed in ticks could be due to higher genotypic diversity in ticks as opposed to in hosts at both the individual and population level.

Significant percentages of individual ticks and hosts were infected by several *rplB* genotypes, indicating potential co-infection by different species. Indeed, 22.6%, 17.8%, and 6.5% of nymphs, chipmunks, and bank voles, respectively, were co-infected by different *rplB* genotypes; 42.3%, 35.6%, and 35.7% of ticks, chipmunks, and bank voles, respectively, were co-infected by different *ospC* genotypes (Table 1).

### Genotype groups and phylogenies

**Figure 1:**
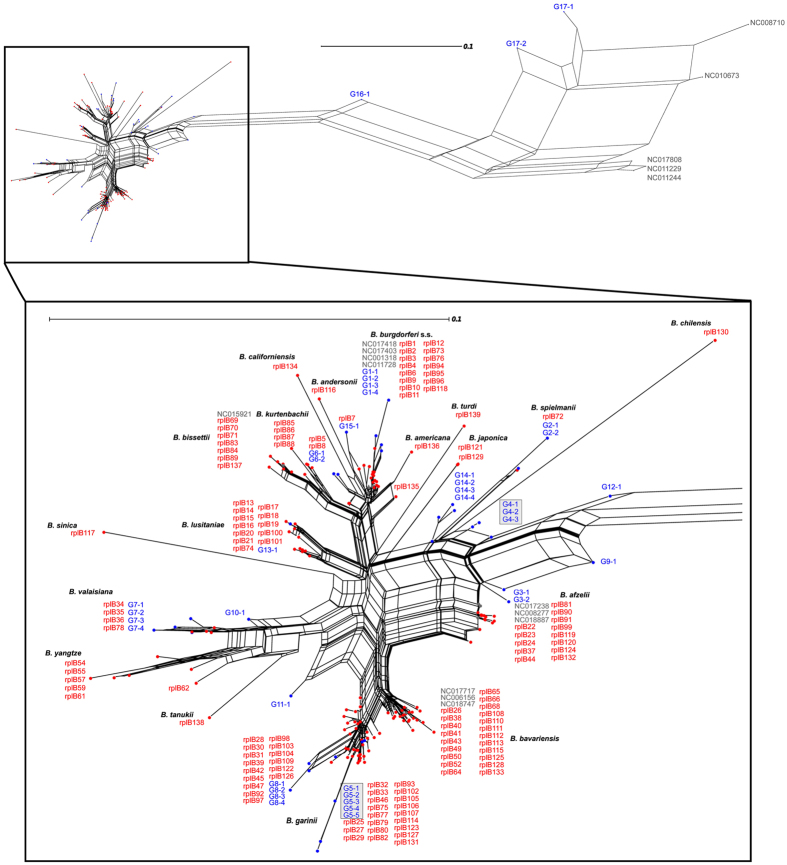
Phylogenetic network of *rplB* sequences. The network was built using (i) consensus sequences of genotypes identified in tick and host individuals sampled in the Sénart Forest for this study (in blue) and (ii) *rplB* reference sequences for members of the *B. burgdorferi* species complex and relapsing-fever spirochetes (in red and gray). All genotypes with the same prefix (e.g., G6) were empirically assigned to the same genotype group (GG) because of their close relationships. GGs that included strains isolated from humans are framed by a gray square.

**Figure 2:**
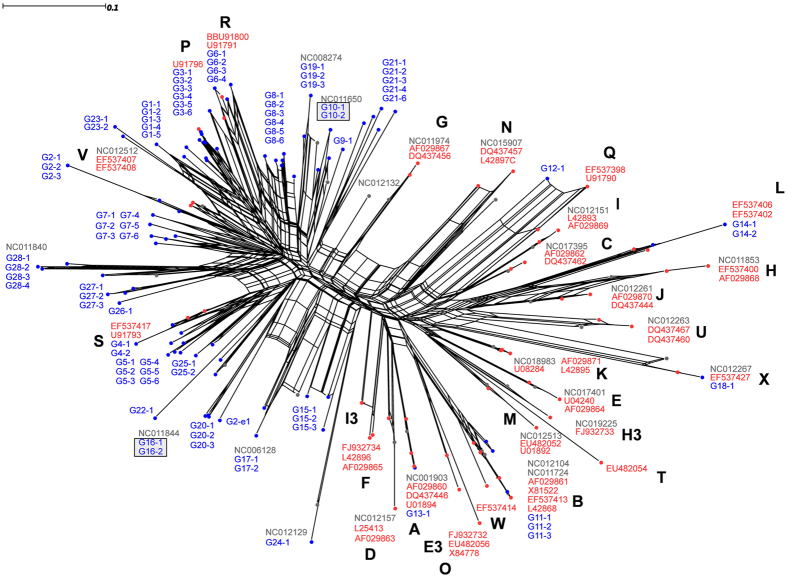
Phylogenetic network of *ospC* sequences. The network was built using i) consensus sequences of genotypes identified in ticks and host individuals sampled in the Sénart Forest for this study (in blue) and ii) available *ospC* reference sequences for members of the *Borrelia burgdorferi* species complex (in red and gray). The capital letters reference individual *ospC* group described in previous studies^18^,^22^,^25^,^26^. All genotypes with the same prefix (*e.g.*, G21) were empirically assigned to the same genotype group (GG) because of their close relationships. GGs that included strains isolated from humans are framed by a gray square framed by a grey square.

Using phylogenetic networks, we were able to empirically delineate groups of closely related genotypes: genotype groups (GGs). A total of 17 and 28 GGs were delineated based on the *rplB* and *ospC* markers, respectively (Figs 1 and 2). We calculated the percentages of individual ticks and hosts that were infected by the different GGs (Supplementary Table S1).

The *rplB* neighbor-net network showed that many members of the *B. burgdorferi* species complex infect ticks and small mammals in the Sénart Forest (Fig. 1): *B. burgdorferi s.s.—rplB* GG G1; *B. spielmanii—rplB* GG G2*; B. afzelii—rplB* GGs G3 and G4; *B. garinii—rplB* GGs G5 and G8; *B. valaisiana—rplB* GGs G7 and G10; and *B. lusitaniae—rplB* GG G13. New genotypes, which did not cluster with any known sequences, were also found. More specifically, *rplB* GGs G6, G11, G12, and G14 could not be assigned to any known species. However, all the GGs observed in small mammals were also observed in ticks. The *rplB* GGs G1 and G4, which are associated with chipmunks^17^, were observed in 93 and 121 nymphs, respectively. The *rplB* GG G3, which is associated with bank voles, was observed in just 13 nymphs. Several GGs occurring in nymphs were not found in the hosts studied. The *rplB* GGs G7 and G8, which were almost never observed in any of the three rodent species sampled, occurred in 49 and 27 nymphs, respectively. Furthermore, *rplB* GGs G16 and G17 were found in 49 nymphs and grouped closely with sequences related to the relapsing-fever spirochetes; they were also situated near the two strains of *B. miyamotoi*, FR64b and LB-2001.

The phylogenetic network of *ospC* genotypes displayed greater genetic diversity than did the *rplB* phylogenetic network; it also displayed a star-like structure (Fig. 2). Of the 27 genotypes listed for the *B. burgdorferi* s.s. species, which are annotated using capital letters^18^,^22^,^25^,^26^, 7 were present in our samples. Twelve of the GGs observed in this study were only found in nymphs (Table S1). The *ospC* GGs most frequently found in ticks were those carried by chipmunks (e.g., *ospC* G1, G10, G11, and G14). Conversely, *ospC* GGs G6, G7, and G21 were each found in more than 35 nymphs but rarely to never in small mammals. Additionally, *ospC* GGs G3 and G8 were observed in 55 and 37 bank voles, respectively, but only rarely in nymphs (12 and 36, respectively).

One of the human strains, IBS18, was assigned to *B. garinii* based on analyses of its *rplB* sequences, which were most similar to those of *rplB* GG G5. The other human strain, IBS42, was assigned to *B. afzelii* based on its *rplB* sequences, which were most closely related to those of *rplB* GG G4. The *ospC* sequences for IBS18 and IBS42 were most similar to *ospC* GG G16 and *ospC* GG G10, respectively.

### Infection group patterns

**Figure 3:**
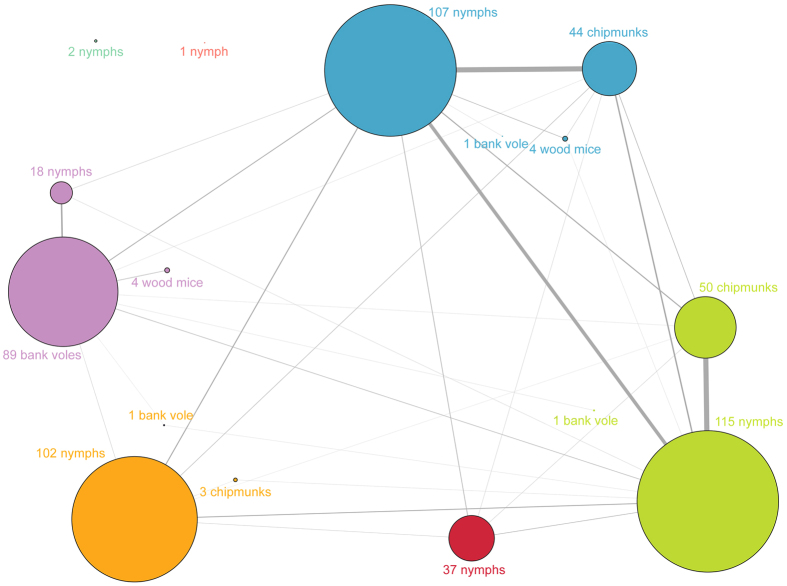
Graphical representation of infection group composition. The graph includes all individual ticks and hosts for which sequences found in at least one of the *rplB* or *ospC* genotype groups (GGs) were obtained. Distinct communities of individuals displaying similar infection patterns (*i.e.*, infection groups, or IGs in the text) were identified using a “greedy approach”; they are graphed in different colors and can be composed of one or more ticks or hosts. Link thickness is proportional to the number of genotypes shared by the individuals of metagroup pairs.

**Figure 4:**
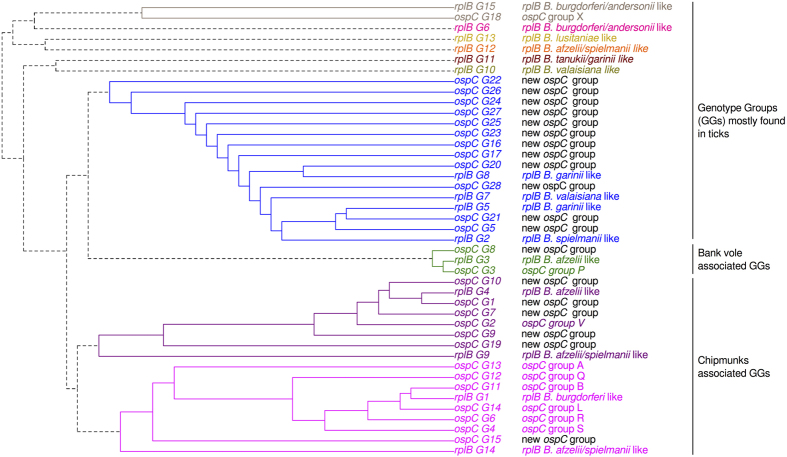
Genotype group association patterns. The dendogram shows the genotype groups (GGs) found using *rplB* and *ospC* sequences detected in tick and host individuals; the *B. miyamotoi rplB* groups G16 and G17 were excluded. Communities of frequently co-occurring GGs within individuals were identified using a greedy approach. Each color of the dendogram corresponds to one of these communities and constitutes a set of genotype groups (SGG). The phylogenetic relationships between GGs and genotypes previously described in the literature are summarized in the next column after GGs’ labels: *rplB* GGs were associated with their *Borrelia* species and *ospC* GGs to previously described genetic groups^18^,^22^,^25^,^26^. The third column summarized species in which the GGs were found.

Using a metagraph, we examined grouping patterns for ticks and hosts based on shared GGs. This analysis showed that seven groups of individuals (*i.e.*, infection groups–IGs) could be distinguished from each other based on shared diversity patterns (Fig. 3). Two IGs were mainly composed of chipmunks (50 versus 44), ticks (115 versus 107), and a few other small mammals (1 bank vole versus 4 wood mice). The chipmunks and ticks were mainly infected by *rplB* GGs G4 and G1, respectively; the GGs appear to be associated with *B. burgdorferi* s.s. and *B. afzelii* (Fig. 1). An analysis of the sets of genotype groups (SGGs) revealed that many *ospC* GGs were also found in these individuals (Fig. 4). A third IG was composed of 89 bank voles, 4 wood mice, and 18 ticks. Two other IGs, which were strongly represented in our samples, were mostly composed of ticks (102 versus 37). The first was predominantly composed of individuals carrying *rplB* GGs G5, G7, G8, and G10, which are closely related to *B. garinii* and *B. valaisiana* sequences (Fig. 1). The other was composed of ticks infected by *rplB* GG G17, which shares similarities with the *B. miyamotoi* sequences. The main *ospC* GGs in these groups are shown Fig. 4. The two last IGs occupied a distinct area of the metagraph and only comprised 2 nymphs and 1 nymph, respectively.

### Spatial distribution of infection groups

To gain further insight into the spatial distribution of infection patterns within Sénart Forest, we mapped the different IGs to which ticks belonged based on tick sampling locations (Supplementary Fig. S4). Further statistical analysis showed that most of the IGs were homogeneously distributed across the forest (data not shown).

### Host contributions to tick infection

One of our aims was to estimate the relative contributions of chipmunks, bank voles, and non-sampled hosts to the numbers of ticks infected by *B. burgdorferi* s.l. genotypes. We began by identifying SGGs in a dataset from which the two *B. miyamotoi rplB* GGs, G16 and G17, were excluded. GGs for both genes did not randomly infect ticks and hosts because 10 SGGs were found when co-occurrence patterns were analyzed (Fig. 4). First, the *B. afzelii rplB* GG G3 is associated with both *ospC* GGs G3 and G8, which are mainly found in ticks and/or bank voles. *B. spielmanii, B. garinii*, and *B. valaisiana rplB* GGs G2, G5, G7, and G8 seemed to occur in the same individuals and were associated with numerous *ospC* GGs only found in ticks (Supplementary Table S1). The third and fourth SGGs grouped together *rplB* GGs G4 and G9 (*B. afzelii*) and *rplB* GGs G1 and G14 (*B. burgdorferi* s.s. and unassigned species) with *ospC* GGs that were mainly found in ticks and/or chipmunks. The *rplB* GG G15 grouped with a single *ospC* GG—*ospC* G18—whereas *rplB* GGs G6, G10, G11, G12, and G13 did not cluster with any other GGs identified in the dataset. To estimate the relative host contributions to tick infection, we compared our simulated data to the observed frequencies of these 10 SGG combinations. We determined the mean values of parameters of interest from posterior distributions (Supplementary Fig. S5). The estimates of the contribution parameters were *α* = 51.0% (chipmunks), *β* = 3.7% (bank voles), and *γ* = 45.3% (non-sampled hosts).

## Discussion

High-throughput sequencing methods offer a new way to characterize the transmission cycles of vector-borne multihost pathogens. In this study, we used them to explore the occurrence patterns of *B. burgdorferi* s.l. pathogens in tick vectors and small mammal hosts; two markers were exploited. Tick vectors displayed a higher level of pathogen genotype diversity than did the hosts. Our analysis of genotype distributions among ticks and hosts allowed us to identify transmission cycles involving sampled and non-sampled hosts. We found large differences in the relative contributions of sampled and non-sampled hosts to tick infection. In particular, this study underscored the importance of taking co-infection patterns into account to better understand the genetic structure of pathogen populations and to link these patterns with the way in which pathogens spread and are maintained in host communities.

### Ticks support parallel transmission

Network analyses based on the distributions of pathogen genotypes revealed the presence of seven distinct infection groups (IGs) in the Sénart Forest; one involved a *Borrelia* species not in the *B. burgdorferi* complex: *B. miyamotoi*. This finding concurs with the results of a previous study, which discovered that two small mammal reservoir species in this forest carried distinct sets of *B. burgdorferi* s.l. genotypes^17^. These IGs probably correspond to different transmission cycles. This result joins with other observations that certain species in the *B. burgdorferi* complex are clearly associated when they occur in *I. ricinus* nymphs; these associations are thought to be shaped by host infection patterns^19^. Our study also shows that network analysis and metagraphing are promising tools for untangling transmission cycles. While they have already been used to study interactions among ticks, vectors, and host species in a meta-analysis^27^, they can also be utilized to study transmission cycles at smaller ecological and evolutionary scales. However, their sensitivity to various statistical and biological constraints has not yet been determined. This would be helpful to evaluate some of the hypotheses that might explain the existence of two independent IGs in chipmunks (*Tamias sibiricus*): i) there is competition among *B. burgdorferi* genotypes within hosts^28^; ii) bottlenecks during host-to-tick transmission could result in low levels of co-infection within vectors because of genetic drift (even if the mean number of genotypes per tick is not lower from the mean number of genotypes per host in this study); iii) other host species carrying only a subset of chipmunk-like GGs are involved; and iv) there is a lack of statistical power because infection and co-infection levels are too low.

### Involvement of non-sampled hosts in transmission cycles

Our study demonstrates that, at the scale of the entire Sénart Forest, the diversity of *B. burgdorferi* s.l. strains found in ticks is higher than that found in small mammals for both *rplB* and *ospC*. This result is congruent with other studies based on *ospC* genotypes^26^ but also based on other markers such as the 5S-23S rRNA intergenic spacer region^29^. This pattern may be explained by several non-exclusive hypotheses. It might be that some genotypes are limited in their ability to infect hosts but are able to circulate among ticks due to co-feeding^30^. However, it is more likely that most of the genotypes identified in ticks are only maintained in host species that we did not sample. Our examination of sets of genotype groups (SGGs) and infection groups (IGs), along with our comparison of the genotypes we characterized with sequences from public databases, allowed us to propose candidate hosts. For instance, birds are likely involved in the circulation of some of the observed genotypes because one SGG included *B. garinii* and *B. valaisiana* genotypes (*i.e.*, GG5, GG8, and GG7), which would explain why the associated IG included only ticks. Indeed, in Europe, *B. garinii* and *B. valaisiana* are usually found in avian reservoirs (reviewed in Margos *et al.*^31^). Four bird species have been found to contribute to tick infection in the Sénart Forest: the common blackbird, *Turdus merula*; the European robin, *Erithacus rebecula*; the song thrush, *Turdus philomelos*; and the winter wren, *Troglodytes troglodytes*^32^. However, the genotypes involved have not been characterized yet and there is an evident lack of information about their ability to infect humans. Indeed, most of the *ospC* genotypes that were shown to be associated with Lyme disease belong to *B. burgdorferi* s.s.^18^,^22^, whereas *B. afzelii* and *B. garinii* play a significant role in European and Asian Lyme epidemiology^33^.

### Contribution of hosts to transmission cycles

Quantifying the contributions of different host species to the circulation of a given pathogen is crucial as it highlights potential sources of infection. It could help inform control efforts that focus on mitigating human health risks through the modification of host communities. The statistical model developed here allowed us to evaluate the relative contributions of sampled and non-sampled host species to the circulation of the *B. burgdorferi* species complex in ticks. With regards to sampled host species, bank voles appeared to contribute less to tick infection than did chipmunks (~4% versus ~51%, respectively). Not surprisingly, the two species have different tick infestation rates—the mean number of nymphs observed on chipmunks is 20, which is 25 times more than on bank voles^34^. This could mean that chipmunks are more likely to be exposed to the bacteria and is at greater risk of infection and co-infection. However, since chipmunks colonized the Sénart Forest between 1970 and 1990^35^, it is possible that the species invested maximal resources in reproduction -benefiting to establishment- to the detriment of its immune system^36^. As a result, chipmunks may have become more vulnerable to *Borrelia* infections than bank-voles^37^. Nevertheless, truly estimating the two species’ reservoir capacities, and understanding the potential roles played by other hosts in pathogen circulation, would require much more ecological data^6^. Setting aside potential sources of error in estimating contributions, non-sampled hosts, mostly birds as hypothesized above, appear to make a strong contribution (~45%) to the circulation of *B. burgdorferi* s.l. genotypes in the Sénart Forest. This finding illustrates the complexity of studies examining the transmission cycles of multihost pathogens in the wild. This percentage fits with what has been found during previous modeling studies that attempted to estimate the contribution of non-sampled hosts to *B. burgdorferi* circulation in the US^20^,^21^. However, it is necessary to point out that our models differ in an important way. Previous models only established an upper limit for the potential contribution made by non-sampled hosts using a threshold-based constraint, an approach that could result in parameter overestimation. In contrast, we sought to maximize the potential contributions of sampled hosts using a sequential modeling approach that provided a conservative estimate of the contribution of non-sampled hosts.

### Transmission cycle structure and zoonotic risk

Based on the genotype distribution patterns among hosts and vectors, the transmission of *B. burgdorferi* s.l. is significantly structured in the Sénart Forest. Recent work on the *Borrelia* populations that infect invasive gray squirrels has revealed a less structured situation^38^; gray squirrels host all the *Borrelia* species that circulate in the United Kingdom. The difference between these two results suggests that *Borrelia* transmission cycles depend on local conditions. This dependency may lead to the existence of a spatial co-evolutionary mosaic in which the heterogeneity of interactions between host-pathogen populations may be more or less favorable pathogen host-range shift or expansion^39^. This idea is of particular interest in the context of zoonotic risk because the ability of multihost pathogens to perform host shifts could influence their ability to persist in changing host communities, spread in new environments, and cause human infections. In the *B. burgdorferi* species complex, only some genotypes tend to cause human infections^22^,^23^. The risk of human Lyme borreliosis thus depends on the frequency of potential zoonotic lineages. The two genotypes obtained from humans infected while in the Sénart Forest were related to genotypes associated with i) non-sampled hosts, which were probably birds and ii) chipmunks. As these transmission cycles have been found to greatly contribute to tick infection, they result in a risky epidemiological situation where frequent genotypes can cause human disease. The *ospC* GGs G13 and G11, which were associated with the two human infections, were found in 5 and 22 chipmunks and 0 and 43 nymphs, respectively. They are closely related to *ospC* major groups A and B, which have been defined by Seinost and colleagues^26^ as alleles of the *B. burgdorferi* species complex that are associated with chronic Lyme disease. These findings highlight the importance of focusing future research on potential correlations among genotype frequencies in ticks and their relevance for human health risks.

## Conclusion

This study has demonstrated that refining molecular and methodological techniques allows us to identify co-occurring genotypes and thus enhance our understanding of the transmission cycles of multihost pathogens. When combined with multidisciplinary approaches, these tools will help us untangle the epidemiology of numerous zoonotic pathogens.

## Materials and Methods

In this study, we first sampled ticks. Then, we identified individuals infected with *B. burgdorferi* s.l. or related *Borrelia* genotypes and sequenced two key bacterial genes. Genotypes at each locus were identified and compared with those previously identified in small mammals^17^. Further analyses were then conducted, which are summarized in Supplementary Fig. S1.

### Tick sampling and *Borrelia* sequencing

#### Ethics statement

Our study did not involve the sampling of any endangered or protected species. According to French law, collecting ticks and carrying out field work at our study site, the Sénart Forest, does not require any specific authorizations. Nevertheless, tick sampling was supervised by employees of the French National Forests Office, which is responsible for managing the Sénart Forest. We obtained two strains isolated from humans who were naturally infected while in the forest; these were provided by the French National Center for *B. burgdorferi* Research (Centre National de Référence [CNR] des *Borrelia*) located in Strasbourg.

#### Tick sampling and selection

In 2011, we sampled *I. ricinus* ticks in the Sénart Forest (3200 ha; 48°40′N, 02°29′E), located near Paris (France), by dragging a large piece of cotton fabric (flag) across the vegetation and leaf litter. A detailed description of our sampling methodology is provided in Vourc’h *et al.*^40^.

Ticks were collected from 220 of the 273 forest stands. Wherever possible, in each forest stand, the flag was dragged along two transects composed of 8 sampling units of 10 m^2^ each (1 × 10 m); the transects were separated from each other by 20 m. At least one tick was collected from 413 of the 440 transects sampled^40^. Ticks from each transect were pooled by developmental stage (*i.e.*, larvae, nymphs, and adults): they were placed in a tube containing 70% ethanol and stored until further analyses could be conducted. From among the 19,546 nymphs collected, 3,903 individuals were selected at random, which resulted in a sampling scheme where there was at least one tick representative for each transect. We focused exclusively on nymphs since larvae are assumed to be infection free and adult ticks have likely already fed on different host species, which would complicate the analyses.

For each tick, DNA was extracted using the ammonia-based protocol described by Humair *et al.*^41^. We then looked for the presence of *Borrelia* DNA in these extracts using quantitative PCR. Two primers were used to amplify part of the *flaB* gene: FlaB_outF 5′-ATATAACCAAATGCACATGTT-3′, and FlaB_inR 5′-ACATTAGCWGMATAAATATTTACAG-3′. They were developed taking into account the diversity of the species complex^42^. Amplification was performed using the SsoAdvanced Universal SYBR Green Supermix kit and the CFX Touch Real-Time PCR Detection System (Biorad, Hercules, California, USA). The total reaction volume was 20 μl and included 10 μl of 2X mix, 2 μl of each primer at 10 μM, 1 μl of H2O, and 5 μl of DNA template solution. Amplification started with an initial denaturation step of 2 min at 98 °C, which was followed by 50 cycles that included a denaturation step of 5 s at 98 °C and an annealing/elongation step of 30 s at 60 °C; fluorescence was recorded for each cycle.

#### Sample characterization

One of the study aims was to conduct an in-depth assessment of the genetic diversity present within infected ticks. To this end, we focused our analyses on the 488 nymphs that were found to be infected by *Borrelia*. We also examined *B. burgdorferi* s.l. isolates (IBS18 and IBS42) that were cultured from two humans infected while in the Sénart Forest. We characterized the genetic diversity of *B. burgdorferi* s.l. genotypes at two different loci, as in a previous study^17^. In short, we focused on partial sequences of the housekeeping gene *rplB* and the infection-related gene *ospC*. Markers were independently amplified via semi-nested PCR for each DNA sample using the Promega GoTaq kit (Madison, Wisconsin, USA) and sample-specific tagged primers (Sigma-Aldrich, Saint-Louis, Missouri, USA). Equal amounts of the purified *rplB* and *ospC* PCR products were then mixed together and sent to GATC-Biotech (Konstanz, Germany) for 454 sequencing on the GS FLX + System (three-eighths of a plate).

### Sequence analysis

#### Rodent sequences

Another study aim was to characterize the transmission cycles of *B. burgdorferi* s.l. genotypes through vectors and hosts. Thus, we combined the *rplB* and *ospC* sequences obtained from nymphs in this study with those obtained from 228 small mammals in a previous study^17^. Among these small mammals were 125 Siberian chipmunks (*Tamias sibiricus barberi*), 93 bank voles (*Myodes glareolus*), and 10 wood mice (*Apodemus sylvaticus*). All the analyses discussed below involved both the nymph and small mammal sequences.

#### Sequence assignment, trimming, and alignment

Using the method described in Jacquot *et al.*^17^, raw sequences were assigned to their respective nymphs or individual hosts and target loci using a BLASTN approach^43^. The tag and the target sequence for each raw read were compared, respectively, to a list of the oligonucleotides used to tag the sequences obtained from each sample and a list of the different representative sequences of the target loci extracted from the *B. burgdorferi* MLST database (http://borrelia.mlst.net)^12^ and GenBank^44^. We only used the first 500 bp of the sequences in subsequent analyses, and sequences shorter than 350 bp were removed from the dataset. For each individual, we aligned the remaining sequences for each locus using K-Align^45^.

#### Genotype delineation and phylogeny

The main disadvantage of high-throughput sequencing methods, as compared to Sanger sequencing, is the high base-calling error rate. When studying diversity, base-call errors can be problematic because they result in low-frequency variants that are in fact artifacts. To circumvent this problem, genotypes were delineated according to two successively applied distance-based nearest-neighbour classifications: i) one within individual samples and ii) one among individual samples^17^. Based on each marker’s diversity pattern and for the both classifications, divergence thresholds were established. We chose thresholds of three and four nucleotide sites for *rplB* and *ospC*, respectively, to both limit the number of genotypes and use a resolution level that did not affect the results of further analyses. Genotypes represented by a limited number of sequences (less than four) were excluded from our analyses.

To verify that the accumulation of sequencing errors was not a major driver of genotype delineation and investigate the degree to which sequencing effort affected the number of genotypes delineated from classifications, a rarefaction analysis on the number of delineated genotypes was performed. It was based on raw sequence sampling at the whole-dataset scale with comparable sequencing efforts for ticks and hosts (same mean number of sequences per individual in both ticks and hosts). Here, we intended to compare the total number of genotypes found in ticks, in hosts, and in both ticks and hosts. For each of the 1000 sequence re-sampling of both genes, we computed the average number of delineated genotypes within individuals and the variance of the number of genotypes observed among individuals.

We then used a phylogenetic approach to examine genotype diversity in greater detail. For each locus, consensus sequences and reference sequences were aligned with each other using K-Align^45^. Phylogenetic searches were performed employing a maximum-likelihood approach and using PHYML^46^. The most appropriate evolutionary model was chosen for each alignment based on the Akaike Information Criterion^47^, which was calculated using the APE library in R^48^. Then, the alignments were used to construct distance matrices using PAUP* 4.0 b10^49^; the best model for each locus was chosen (*i.e.*, TN93 + I + G for *rplB* and GTR + I + G for *ospC*). Substitution rate matrices were estimated via maximum likelihood, and we assumed empirical nucleotide frequencies. According to the results of the PHYML analysis, the shape parameters of the gamma distributions were fixed at 0.663 for *rplB* and 0.958 for *ospC*, and the proportion of invariable sites were fixed at 0.410 for *rplB* and 0.233 for *ospC*. Then, phylogenetic networks were obtained for both loci using the Neighbour-Net method^50^ and SPLITSTREE4^51^. Networks provide an opportunity to visualize ambiguities in relationships that can have been cause by recombination events involving the studied locus.

#### Delineation and co-occurrence of genotype groups within nymphs and host species

Using the networks, we were able to empirically delineate groups of closely related genotypes, which we called genotype groups (GGs). GGs were numbered according to a previously established nomenclature system^17^.

First, we aimed to identify the different transmission cycles involving the *B. burgdorferi* s.l. GGs we observed in nymphs and small mammals. We did so by examining GG co-occurrence patterns. We built a contingency table that described the occurrence of GGs across individuals (*i.e.*, an incidence matrix). This m*n matrix, where *m* was the number of individual ticks and hosts and *n* was the number of GGs, was weighted such that the sum of each row, which corresponded to a single individual, equaled one. After describing the presence/absence of different GGs among different individuals, we created an m*m co-occurrence matrix that described the number of GGs shared by pairs of individuals. This matrix was obtained by multiplying the incidence matrix by its transpose. Next, we evaluated which individual ticks and hosts showed similar patterns of infection by GGs to identify infection groups (IGs). These sets of highly connected individuals (referred to as communities in graph theory) were defined using a “greedy” approach^52^. This approach optimized the classification of individuals to IGs in the following ways: i) by maximizing the modularity index that reflects the ratio of GGs shared among individuals both within IGs and among IGs and ii) by assigning individuals to the smallest number of IGs possible. We could then calculate the proportion of individual nymphs and hosts assigned to each IG. Finally, we summarized all this information by building a metagraph that distinguished between the different IGs and that revealed the ticks and small mammal species found within each IG.

Similarly, with the aim of highlighting multilocus associations (*i.e.*, GGs at both markers that appeared in the same hosts), we created an n*n co-occurrence matrix describing the number of hosts and ticks that shared pairs of GGs. This matrix was obtained by multiplying the incidence matrix and its transpose. The “greedy” clustering algorithm described above^52^ allowed us to illustrate the associations among GGs— meaning the sets of genotype groups (SGGs) infecting the same individual nymphs and hosts—with a dendogram. Due to the frequent co-infections within individuals linkage disequilibrium between loci can not be measured, but this dendogram provides an alternative picture of this linkage.

These analyses were performed using the igraph library in R^53^. The similarity matrix was obtained from the incidence matrix using the graph.incidence and the bipartite.projection functions. The classification analyses were performed using the fastgreedy.community function, and the graphical output was produced using the tkplot and dendPlot functions.

#### Spatial distribution of infection groups

To visualize the spatial distribution of IGs at our study site, we built a map using QGIS 2.4. Since nymphs sampled along the same transect were pooled, the exact location for each tick was not known. We therefore had to aggregate data at the centroid of the transects. It was thus possible to observe more than one IG at a given location.

### Contribution model: host contributions to pathogen diversity in ticks

We developed a statistical mixture model to estimate the relative (percentage) contributions made by different hosts to the number of nymphs infected by *B. burgdorferi* s.l. pathogens. We looked at chipmunk-associated GGs, bank-vole-associated GGs, and GGs unassociated with any of the hosts studied to determine if additional, unsampled vertebrate species could be acting as major hosts for *B. burgdorferi* s.l. Such a model could also be used to predict which vertebrate species, within the whole host community, was the likely source of *B. burgdorferi* s.l. infections in humans. An overview of the model is available on Supplementary Fig. S2. Detailed explanations are given bellow.

Our model mixed features of both the “signature-matching” and “inverse-model” approaches (developed by Brisson *et al.*^21^) to deal with the specificities of our data. For instance, we did not have estimates of GG transmission probabilities for each host species-tick pair. These probabilities are required for the inverse model. We therefore based our analysis on the similarity of bacterial genotypes in infected hosts and ticks, as in the signature-matching model. However, we wished to avoid the rule-based assignation of ticks to host reservoirs to infer host contributions—the method used by the signature-matching model. We consequently employed the inverse model’s simulation approach. We used observed SGG presence/absence patterns in ticks and small mammals to fit the model, making it possible to fit computational time constraints and to take into account the information associated with both markers and co-infection patterns.

By definition, the infection patterns of non-sampled hosts (hereafter referred to as the X category) were unknown. To avoid situations in which the X category’s inferred infection patterns completely matched the ticks’ infection patterns, which would have meant that the X category could be the only significant contributor, we simulated tick infection patterns using a series of analyses.

The first analysis aimed to establish a distribution of the contributions of sampled hosts; the X category was excluded. This distribution therefore reflected the maximum potential contributions of transmission cycles involving chipmunks and bank voles. First, using the observed data, we scored individual samples based on the presence/absence of alleles belonging to each SGG. Second, we scored individual samples based on the presence/absence of alleles associated with all possible SGG combinations (*e.g.*, SGG1, SGG1-SGG2, SGG2, SGG2-SGG3, all SGGs, etc.). This allowed us to obtain two vectors—*C*_*iTs*_ and *C*_*iMg*_—that described the observed frequencies of each SGG combination *i* among chipmunks and bank voles, respectively. In each simulation, these vectors were used to calculate expected frequencies of SGG combinations in ticks, *C*_*iTkSim*_, when the X category was excluded and only the variable contributions of chipmunks and bank voles were taken into account. Finally, the respective contributions of chipmunks, bank voles, and the X category to tick infection—*α*, *β*, and *γ*—were generated randomly such that *α* + *β* + *γ* = 1. *C*_*iTkSim*_ was then determined using *αC*_*iTs*_ + *βC*_*iMg*_.

The value of *C*_*iTkSim*_ was simulated one million times and compared to the observed value of *C*_*iTkObs*_ using Euclidean distances. The 10% of contribution values {*α, β, γ*} that resulted in the simulations most similar to the observed data were retained for the second series.

Using the retained {α, β, γ} values, patterns of SGG combination presence/absence in ticks were then characterized: *C*_*iTkSim*_ = *αC*_*iTs*_ + *βC*_*iMg*_ + *γC*_*iX*_. To this end, a *C*_*iX*_ vector was calculated for each simulation in the following manner: i) a frequency for each SGG was drawn from a uniform distribution with a range between 0 and 1 and ii) the frequency of each SGG combination in ticks was calculated based on the frequency of each SGG; it was assumed that SGGs were randomly associated within ticks and that ticks were infected by at least one SGG, which ensured that the sum of SGG combination frequencies equaled 1. The results of 100,000 simulations were then compared to the observed data by calculating Euclidean distances for the simulated and observed values of i) the *C*_*iTkSim*_ and *C*_*iTkObs*_ vectors and ii) the vectors of the differentiation values (Wright’s *F*_*ST*_^54^). The latter were determined for each SGG in ticks versus chipmunks and in ticks versus bank voles. Sets of parameters were retained if the distance was lower than the tolerance threshold, which was defined such that the results of 1% of the simulations were retained.

## Additional Information

**How to cite this article**: Jacquot, M. *et al.* Multiple independent transmission cycles of a tick-borne pathogen within a local host community. *Sci. Rep.* **6**, 31273; doi: 10.1038/srep31273 (2016).

## Supplementary Material

Supplementary Information

## Figures and Tables

**Table 1:** Number of tick and host individuals (chipmunks and bank voles) included in the analysis and their percentages of co-infection by at least two *Borrelia burgdorferi* s.l. and/or of *B. miyamotoi* genotypes.

Species	n*	*rplB*	*ospC*
*Ixodes ricinus* (ticks)	472	22.60%	42.81%
*Tamias sibiricus barberi* (chipmunks)	96	17.78%	35.56%
*Myodes glareolus* (bank voles)	92	6.52%	35.72%

^*^Number of individuals that yielded sequence data.
