# Supplementary material for: Multiple independent transmission cycles of a tick-borne pathogen within a local host community
Source: Sci Rep. 2016 Aug 8;6:31273. doi: 10.1038/srep31273 (PMC4976386; doi:10.1038/srep31273)
Supplement: Supplementary Information [file srep31273-s1.pdf]

Multiple independent transmission cycles of a tick-borne pathogen within a local host community

AUTHORS AND AFFILIATIONS

Maude Jacquot[1, 4, *], David Abrial[1], Patrick Gasqui[1], Severine Bord[1], Maud Marsot[1,5], Sébastien Masseglia[1], Angélique Pion[1], Valérie Poux[1], Laurence Zilliox[2], Jean-Louis Chapuis[3], Gwenaël Vourc'h[1], and Xavier Bailly[1]

1: INRA, UR346 Epidémiologie animale, Saint Genès Champanelle, France
2: CNR des Borrelia, Hôpitaux Universitaires de Strasbourg, 6700 Strasbourg, France
3: MNHN, Centre d'Ecologie et des Sciences de la Conservation, UMR 7204, Sorbonne Universités, MNHN, CNRS, UPMC, CP53, 61 rue Buffon, 75005 Paris, France
4: Present address: Institute of Biodiversity, Animal Health and Comparative Medicine, College of Medical, Veterinary & Life Sciences, University of Glasgow, Glasgow, G12 8QQ, UK
5: Present address: ANSES, Unité Épidemiologie, 14 rue Pierre et Marie Curie, 94706 Maisons-Alfort Cedex, France

SUPPLEMENTARY INFORMATION

LEGENDS

Figure S1. Delineation of genotypes, genotype groups, sets of genotype groups, and infection groups

(A) Using data for each of the two loci, genotypes were delineated based on the raw consensus sequences. (B) Genotype groups (GGs) were empirically delineated using the loci phylogenies, in which closely related genotypes clustered. (C) Infection groups (IGs) were also delineated using a classification approach. They are groups of individuals (ticks and/or mammals) that displayed similar infection patterns. (D) Sets of genotype groups (SSGs) were delineated using a classification approach. They are communities of frequently co-occurring GGs found within individuals.

Figure S2. Overview of the statistical approach used to measure host-species contribution to tick infections

A) In a first step, the frequencies of sets of genotype groups in characterized hosts species, C_{iTs} and C_{iMg}, are weighted according to two contribution parameters (α and β) optimized by approximate Bayesian computation to explain the frequencies of sets of genotype groups in ticks, C_{iTk}. B) In a second step, the difference between the weighted sum of C_{iTs} and C_{iMg} compared to C_{iTk} is used to infer the frequencies of sets of genotype groups (C_{iX}) in unsampled hosts and their contribution to tick infections (γ) during a second round of approximate Bayesian computation.

Figure S3. Rarefaction analyses of *rplB and ospC* sequences

Rarefaction analyses were conducted by resampling 1000 times raw *rplB* (dots and dashed lines) and *ospC* (diamond and solid lines) sequences; sequencing efforts for ticks versus hosts were comparable (same mean number of sequences per individual). Statistics averaged values (using 750 sequences intervals) as well as fitted local regressions were plotted. (A) Number of genotypes delineated within the whole dataset (in black), within ticks (in blue), and within hosts (in red) as a function of the number of resampled *rplB* and *ospC* sequences. (B) Mean number of genotypes per individual tick and host (in black), per tick (in blue), and per host (in red) as a function of the number of resampled *rplB* and *ospC* sequences. (C) Variance in the number of genotypes per individual tick and host (in black), per tick (in blue), and per host (in red) as a function of the number of resampled *rplB* and *ospC* sequences.

Figure S4. Spatial distribution of infection groups

The spatial distribution of the infection groups (IGs) identified in this study is displayed on a map of the Sénart Forest. The map was built by authors using QGIS 2.4. IGs, or communities of individuals with similar infection patterns, were defined using a “greedy” approach. The seven IGs are represented in different colors. The pie charts indicate the relative presence of different IGs on the transects sampled; their size is proportional to the number of ticks they infected.

Figure S5. Distributions of the contribution model parameter values

The distributions of the values of the parameters of interest for the simulations we selected (based on their similarity to the observed data) are plotted. α, β, and γ correspond to the contributions made by chipmunks, bank voles, and non-sampled hosts (the X category), respectively.

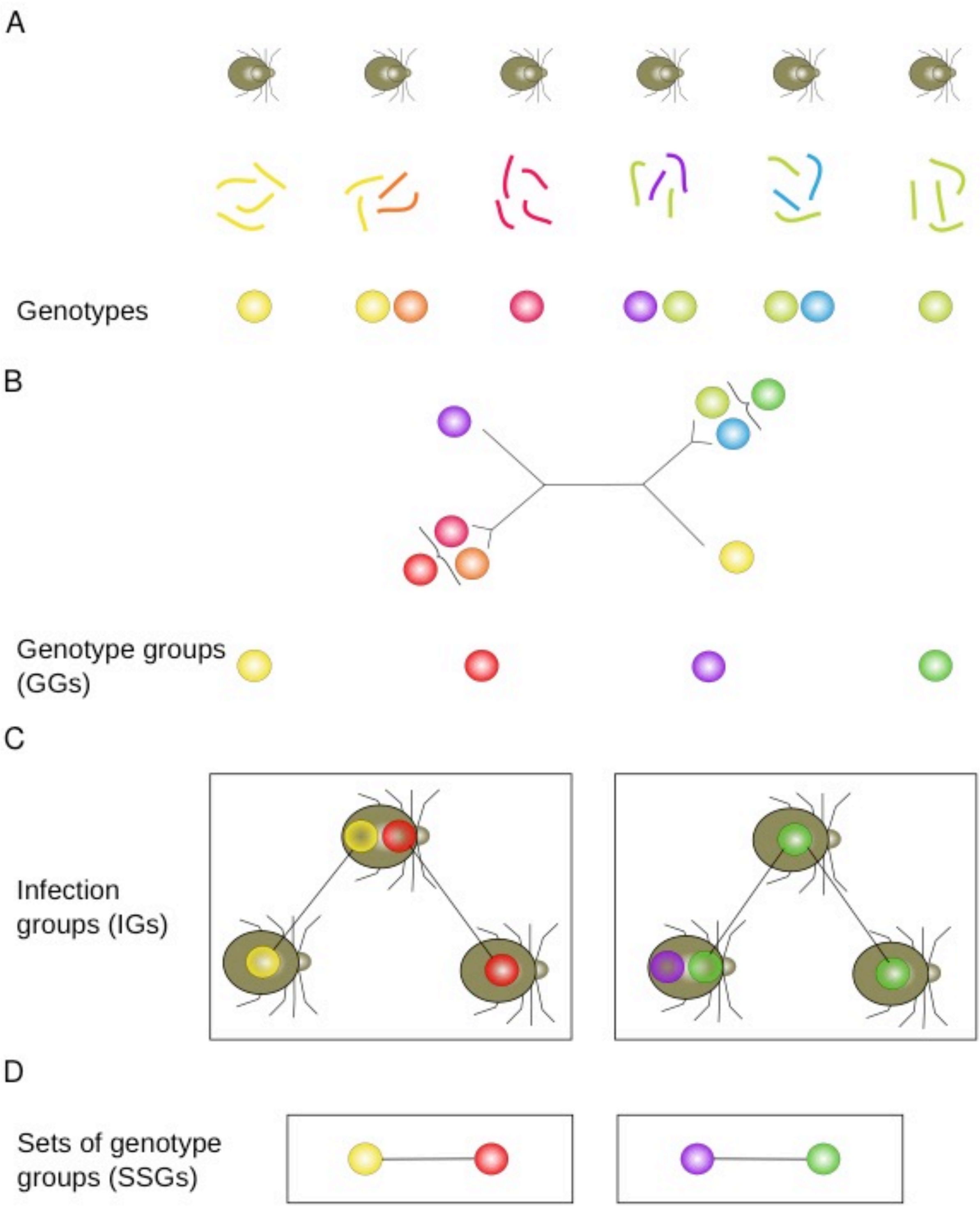

Figure S1. Delineation of genotypes, genotype groups, sets of genotype groups, and infection groups

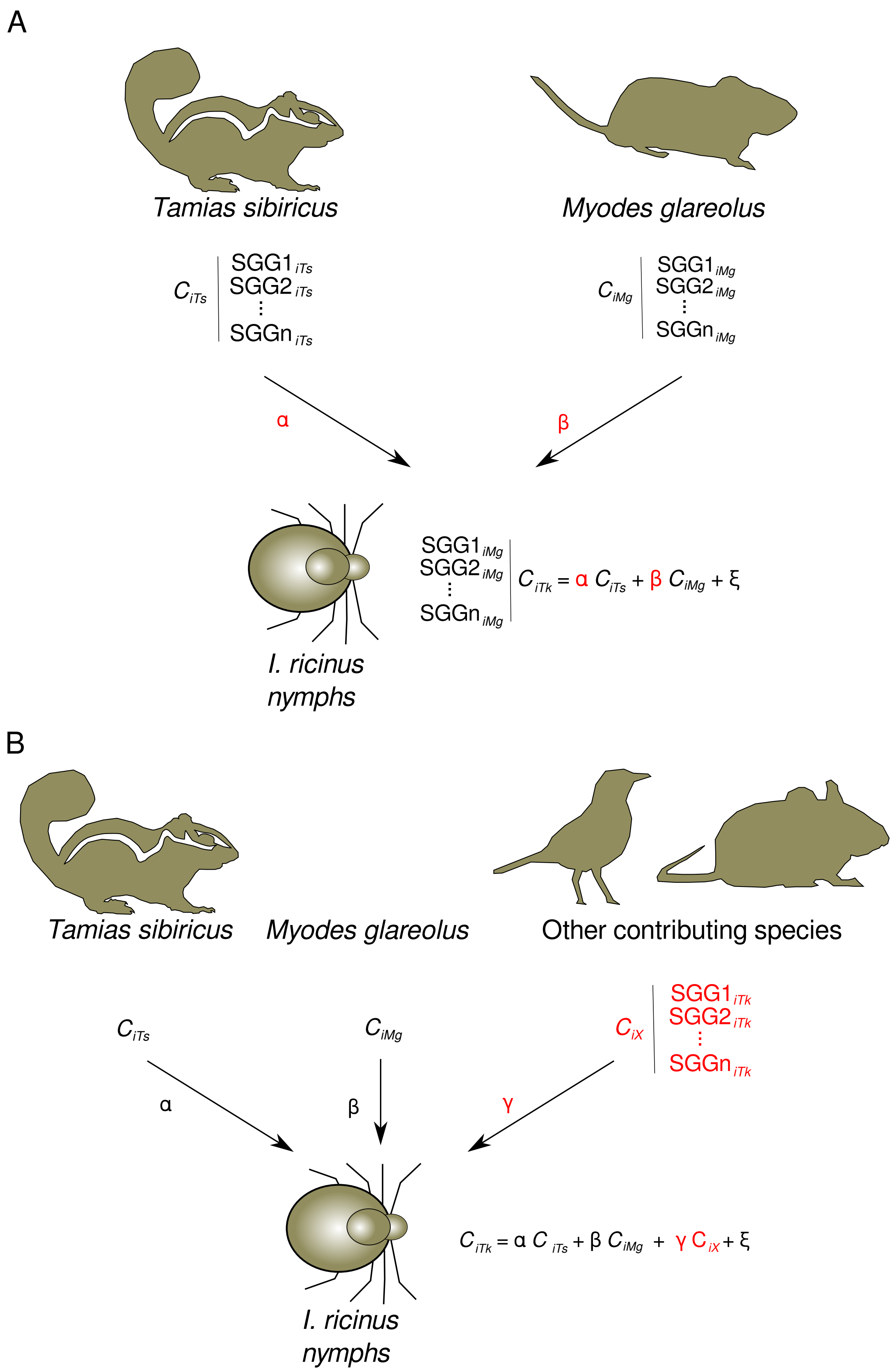

Figure S2. Overview of the statistical approach used to measure host-species contribution to tick infections

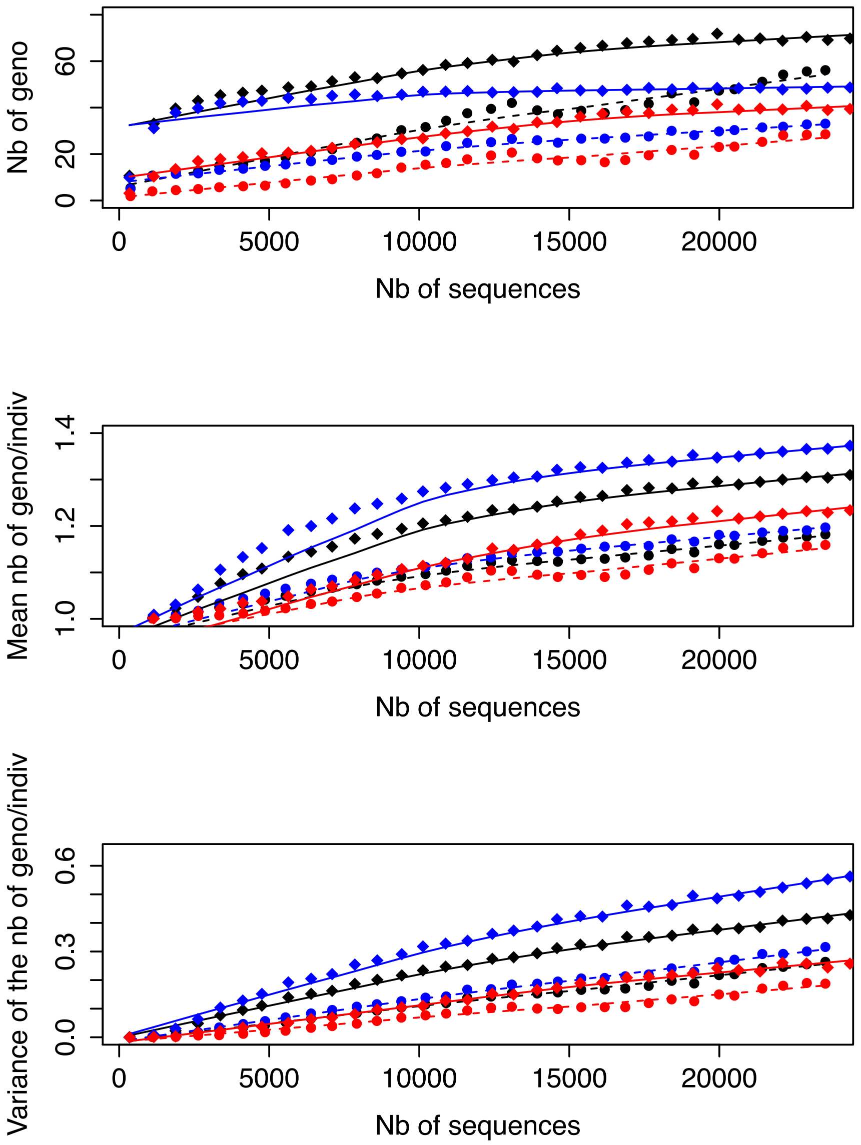

Figure S3. Rarefaction analyses of *rplB and ospC* sequences

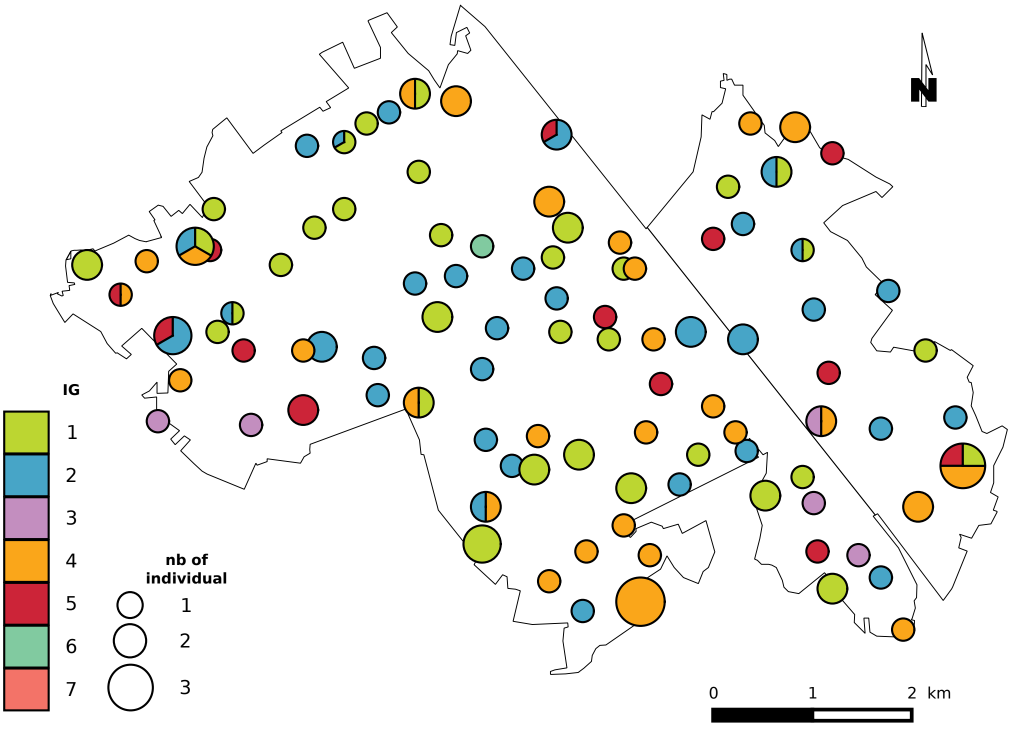

Figure S4. Spatial distribution of infection groups

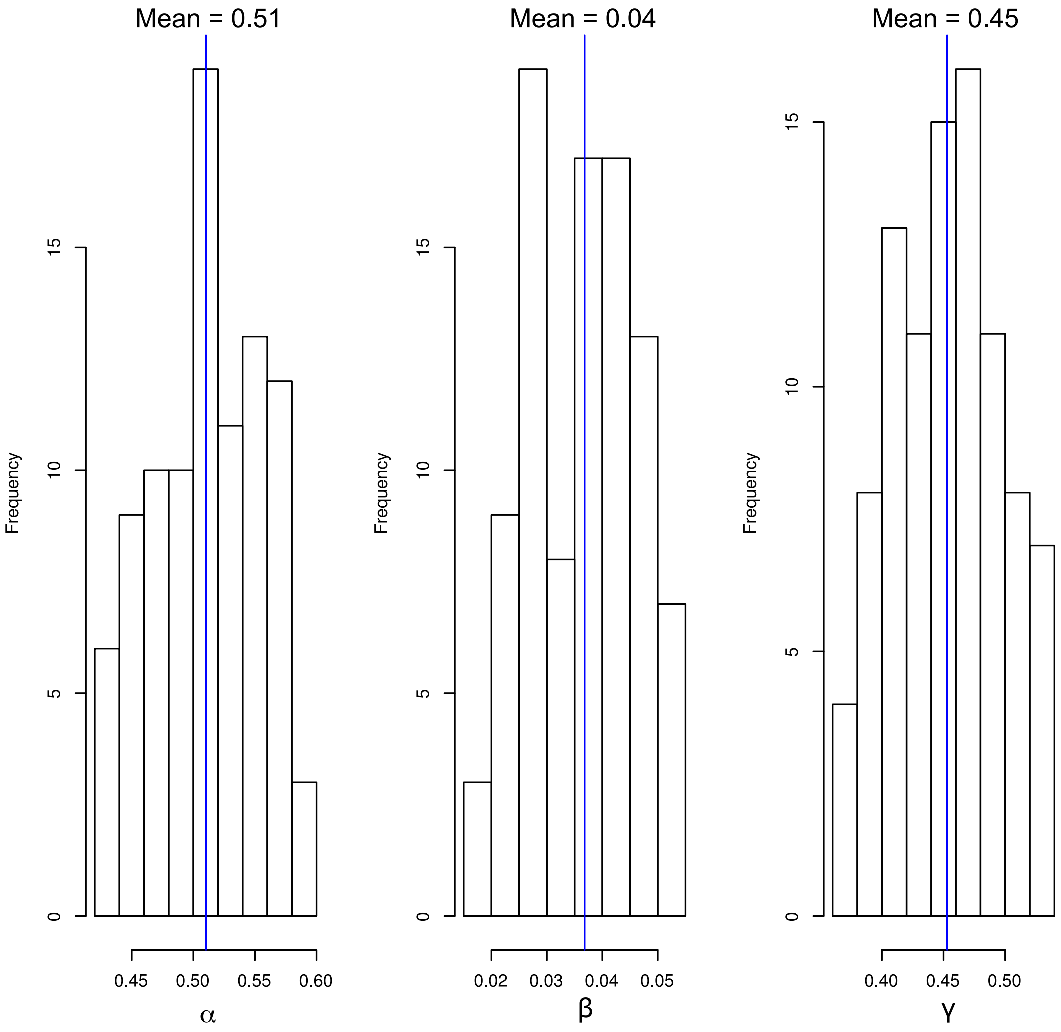

Figure S5. Distributions of the contribution model parameter values

Table S1: Number of ticks, chipmunks, and wood mice infected by each genotype group for the *rplB* and *ospC* loci

Gene	Genotype Group (*ospC* groups nomenclature)	Number of nymphs	Number of chipmunks	Number of bank voles	Number of wood mice
rplB	G1	93	45	1	0
rplB	G2	5	2	0	0
rplB	G3	13	1	90	4
rplB	G4	121	51	1	4
rplB	G5	41	2	1	0
rplB	G6	6	1	0	0
rplB	G7	49	0	0	0
rplB	G8	27	1	0	0
rplB	G9	1	0	0	0
rplB	G10	4	0	0	0
rplB	G11	2	0	0	0
rplB	G12	1	0	0	0
rplB	G13	1	0	0	0
rplB	G14	7	0	0	0
rplB	G15	1	0	0	0
rplB	G16	1	0	0	0
rplB	G17	48	0	0	0
ospC	G1	35	24	3	0
ospC	G2 (group V*)	31	3	0	1
ospC	G3 (group P*)	12	0	55	0
ospC	G4 (group S*)	22	4	0	0
ospC	G5	32	2	1	0
ospC	G6 (group R*)	45	9	0	0
ospC	G7	64	4	0	0
ospC	G8	36	1	37	1
ospC	G9	22	2	0	0
ospC	G10	52	14	0	1
ospC	G11 (group B*)	43	22	0	0
ospC	G12 (group Q*)	11	1	0	0
ospC	G13 (group A*)	0	5	1	0
ospC	G14 (group L*)	38	17	0	0
ospC	G15	20	0	0	0
ospC	G16	9	0	0	0
ospC	G17	5	1	0	0
ospC	G18 (group X*)	3	0	0	0
ospC	G19	8	0	0	0
ospC	G20	14	0	0	0

ospC	G21	38	1	0	0
ospC	G22	1	0	0	0
ospC	G23	11	0	0	0
ospC	G24	3	0	0	0
ospC	G25	8	0	0	0
ospC	G26	2	0	0	0
ospC	G27	5	0	0	0
ospC	G28	16	0	0	0

**ospC* groups as described in previous studies [1,2,3,4]

1 Wang, I. N. *et al.* Genetic diversity of ospC in a local population of *Borrelia burgdorferi* sensu stricto. *Genetics* **151,** 15–30 (1999).

2 Rudenko, N. *et al.* Detection of *Borrelia burgdorferi* sensu stricto *ospC* alleles associated with human lyme borreliosis worldwide in non-human-biting tick *Ixodes affinis* and rodent hosts in southeastern United States. *Appl. Environ. Microbiol.* **79,** 1444–1453 (2013).

3 Seinost, G. *et al.* Four clones of *Borrelia burgdorferi* sensu stricto cause invasive infection in humans. *Infect. Immun.* **67,** 3518–3524 (1999).

4 Brisson, D. & Dykhuizen, D. E. *ospC* diversity in *Borrelia burgdorferi* : different hosts are different niches. *Genetics* **168,** 713–722 (2004).
